# Teliospore mucilage of *Puccinia miscanthi* revealed through the axial imaging of secondary electrons

**DOI:** 10.1186/s42649-021-00064-9

**Published:** 2021-10-22

**Authors:** Ki Woo Kim

**Affiliations:** 1 Department of Ecology and Environmental System, Kyungpook National University, Sangju, 37224 Republic of Korea; 2 Tree Diagnostic Center, Kyungpook National University, Sangju, 37224 Republic of Korea

**Keywords:** Mucilage, *Puccinia miscanthi*, Teliospore

## Abstract

*Puccinia miscanthi* teliospores were observed on the leaf surface of *Miscanthus sinensis* using a field emission scanning electron microscope. Details of teliospore mucilage could be visualized through the axial imaging of secondary electrons for a better understanding of pathogen behavior in rust diseases.

Secondary electrons (SEs) are generated from the primary electron beam-specimen interaction. For scanning electron microscopy (SEM), SEs are collected using either a lateral below-lens Everhart-Thornley (ET) detector or an axial in-lens detector. The two types of SE detectors have pros and cons depending on research objectives. Comparative low-kV SE imaging revealed the fine details of epicuticular waxes on air-dried strawberry leaves through the axial detector (Kim et al. 2009). Meanwhile, the ET detector would be an option for a higher topographic contrast.

*Puccinia* species represent pathogenic fungi causing rust diseases of various plants worldwide. They have several types of spores including teliospores, urediniospores, aeciospores, and basidiospores. Teliospores of *Puccinia* species are commonly observed after chemical fixation and dehydration in a graded series of either acetone or ethanol (Bonde et al. 2015; Morin et al. 1992; O’Keefe and Davis 2015). Although mucilaginous exudates were found on the spore surface, the preparation procedures might have partially removed them. Mucilaginous exudates are associated with host infection and pathogen survival against adverse conditions (O’Keefe and Davis 2015). It is worthwhile to visualize mucilaginous exudates for a better understanding of pathogen behavior in rust diseases.

Rust-infected symptomatic leaves of *Miscanthus sinensis*, eulalia grass, were collected in the field and dried at room temperature for two months (Kim 2015). They were sputter-coated with platinum and observed using a field emission SEM (FESEM) (Supra 55VP; Carl Zeiss, Oberkochen, Germany) at 2 kV with the two types of SE detectors. FESEM revealed the surface details of *Puccinia miscanthi* teliospores using an axial detector (Fig. 1). The spore surface was covered with a netlike substance, mucilaginous exudates. Such characteristics were not so contrasted through the ET detector, compared with those through the axial one (data not shown). Direct imaging without chemical fixation and dehydration was assumed to be effective at preserving spore mucilage. Due to the slight differences in height variations on spore surface, the axial detector was appropriate for low-kV SE imaging. These results suggest the combined use of air-drying spores and low-kV axial SE imaging may provide a basis for visualization of the fine details of mucilaginous exudates on rust teliospores.

**Fig. 1:**
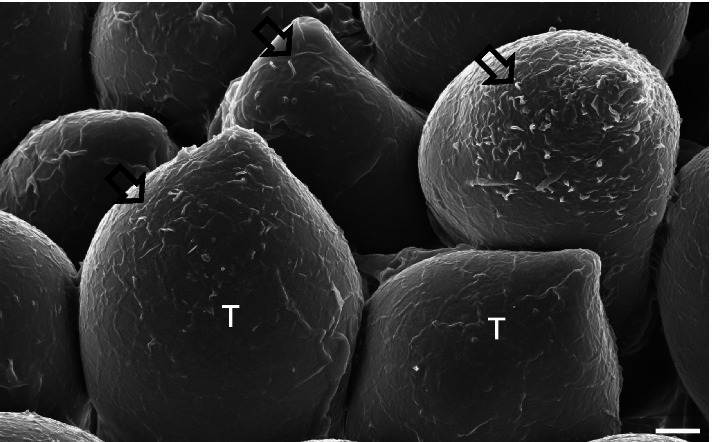
Scanning electron micrograph of *Puccinia miscanthi* teliospores on the leaf surface of *Miscanthus sinensis*. The specimen was imaged using an axial secondary electron detector. Arrows: mucilage. T: teliospore. Scale bar = 2 μm

## Data Availability

Data and materials available on request.
